# Deep learning and multi-omics approach to predict drug responses in cancer

**DOI:** 10.1186/s12859-022-04964-9

**Published:** 2022-11-28

**Authors:** Conghao Wang, Xintong Lye, Rama Kaalia, Parvin Kumar, Jagath C. Rajapakse

**Affiliations:** School of Computer Science and Engineering, Nanyang Technological University, 50 Nanyang Avenue, Singapore, 639798 Singapore

**Keywords:** Cancer, Deep learning, Drug response, Feature embedding, Multi-omics

## Abstract

**Background:**

Cancers are genetically heterogeneous, so anticancer drugs show varying degrees of effectiveness on patients due to their differing genetic profiles. Knowing patient’s responses to numerous cancer drugs are needed for personalized treatment for cancer. By using molecular profiles of cancer cell lines available from Cancer Cell Line Encyclopedia (CCLE) and anticancer drug responses available in the Genomics of Drug Sensitivity in Cancer (GDSC), we will build computational models to predict anticancer drug responses from molecular features.

**Results:**

We propose a novel deep neural network model that integrates multi-omics data available as gene expressions, copy number variations, gene mutations, reverse phase protein array expressions, and metabolomics expressions, in order to predict cellular responses to known anti-cancer drugs. We employ a novel graph embedding layer that incorporates interactome data as prior information for prediction. Moreover, we propose a novel attention layer that effectively combines different omics features, taking their interactions into account. The network outperformed feedforward neural networks and reported 0.90 for $R^2$ values for prediction of drug responses from cancer cell lines data available in CCLE and GDSC.

**Conclusion:**

The outstanding results of our experiments demonstrate that the proposed method is capable of capturing the interactions of genes and proteins, and integrating multi-omics features effectively. Furthermore, both the results of ablation studies and the investigations of the attention layer imply that gene mutation has a greater influence on the prediction of drug responses than other omics data types. Therefore, we conclude that our approach can not only predict the anti-cancer drug response precisely but also provides insights into reaction mechanisms of cancer cell lines and drugs as well.

## Background

Inherent complexity and heterogeneity of cancers make patients with same diagnosis respond differently to anticancer drugs, making cancer treatment difficult and intractable. In order to personalize cancer treatment, it is crucial to know drug responses of cancer patients, based on their molecular and clinical profiles. To address this, extensive patient drug screening is required to discover specific patterns of drug responses. Infeasibility of treating large populations of cancer patients has motivated researchers to collect large scale drug screening data on cancer cell lines. For example, Cancer Cell Line Encyclopedia (CCLE) [1] contains various types of molecular profiling data and the Genomics of Drug Sensitivity in Cancer (GDSC) project [2] contains a comprehensive selection of pan-cancer cell line ($\sim 1000$) drug sensitivity responses to a wide list of anti-cancer drugs ($\sim 200$). The main mission of these projects is to facilitate development of integrated computational models and tools that enable prediction of drug-target interactions and pharmacological responses from cell molecular profiles and drug chemical features.

There are mainly two types of methods for predicting drug responses *in silico* from cancer cell lines: machine learning based approaches and network-based approaches. Machine learning approaches first extract features from multiple molecular measures (gene expression, protein expression, mutation, etc.) and then use classifiers or regressors such as support vector machines [3, 4], elastic-net regression [5], and random forest [6] to make predictions. Recently, deep neural networks have gained popularity in predicting drug responses from cell lines by using molecular descriptors [7, 8]. Network-based methods either build drug-target interaction networks or similarity networks between cell lines and between drug descriptors and then use different network analysis techniques to predict drug responses [9, 10]. However, network-based methods are based on the assumptions that similar molecular profiles and similar drug chemical profiles lead to similar drug responses.

Deep learning methods such as neural networks have the ability to build complex and accurate models by learning from training data and been successful in many application domains [11]. Lately, applications of deep learning have emerged in pharmaceutical research and drug discovery [12]. Liu et al. used two convolutional neural networks, one for processing genomic features of cell lines and another for processing molecular descriptors of drugs, and combined their outputs to predict drug responses [7]. Chiu et al. proposed two deep neural networks, one to process gene expression data and one to process gene mutation data, and then combined two networks to predict drug responses [8]. However, these approaches use multiple neural networks to process different omics data types and then use simple concatenation of features to integrate different networks. There are several challenges when deep neural networks are employed for drug response prediction: (i) huge dimensionality of inputs, (ii) heterogeneity of inputs as data come from different omics platform, (iii) limited number of samples, and (iv) large number of network parameters. In this paper, we present a deep neural network model that addresses some of these issues and is capable of integrating multiple cellular attributes effectively.

CCLE cell lines initially characterized by expression and genomic data have now been expanded to include genetic, RNA splicing, DNA methylation, histone modification, and mRNA expression data [13]. Integration of different molecular or multi-omics features realizing full potential of biological insights from biomolecular interactions pose a huge algorithmic challenge [14]. Due to highly complex nature of mechanisms of cancer, it is difficult to achieve accurate prediction based on a single facet such as genomics solely. Multi-omics techniques where multiple types of omics data such as genomics, proteomics, transcriptomics, and metabolomics have been popular in recent years as they provide more holistic molecular perspective of studied biological systems compared to traditional approaches [15]. Deep learning approaches for integration of multi-omics data are beginning to emerge [16, 17]. Zhang et al. used an autoencoder and k-means clustering to combine gene expression and copy-number variation data to predict subtypes of neuroblastoma [16]. Huang et al. used two neural networks to process mRNA expression and miRNA expression data and combined their outputs to predict survival of breast cancer patients [17]. However, these approaches combine only two types of omics data and use simple mechanisms for integrating omics data. In this work, we propose a neural network that gives a general framework for efficient integration of multiple omics types.

One of the contributions in our work is the integration of the interactome data while processing genomic features. The interactome provides prior knowledge of gene/proteins regulatory and physical interactions. Inspired by [18], we introduce a graph embedding layer for networks processing gene expressions, Copy Number Variation (CNV), mutations and reverse phase protein array (RPPA) data, which incorporates prior information from the interactome, and demonstrate an improvement of performance. Furthermore, in order to obtain insights into the influences of different omics types, we employ an attention layer to efficiently combine network features processing different omics data. By analysing the attention weights, we demonstrate that gene mutation and RPPA data have a stronger impact over the other omics on anti-cancer drug response prediction.

## Results

mRNA expression, mutations, CNV, RPPA expression, and metabolite expressions were used as input to the deep neural network (DNN). Data standardization and noise removal were performed to optimize the training process. For mRNA expression, RPPA and metabolite expressions, if certain expression had zero values across over 95% of the cell lines, it was removed from the dataset. Similarly, for gene mutations and CNV, if certain mutation type had no effect on over 95% of cell lines, it was also eliminated. For omics data using graph embeddings, the embedding layer size was same as that of the input layer. The size of the dense embedding layer for metabolites was 200. For mRNA expression and metabolomics datasets, the dense layer had size 64. For gene mutation and CNV datasets, the hidden sizes were 32. As for RPPA dataset, the hidden size was 128. The attention layer dimension was 110 and output layer dimension was 22. The layer sizes were empirically determined for best performance. We used Adams optimizer [19] to minimize the means square error loss at a learning rate of 0.001. The dropouts were also used: dropout rate was 0.2 for mRNA expressions, mutations, and metabolites, and 0.4 for CNV and RPPA. These hyperparameters were tuned to reduce the complexity of our model in the aim of avoiding overfitting. The simulations were performed on Google Colab’s cloud machine with 32GHz CPU with GPU capabilities. When graph embedding layer was used, the convergence was faster in about 200 epochs but with dense embedding layers, the convergence took about 1000 epochs. Three-fold cross-validation was implemented for all the experiments and the results shown in Table 1 and Table 2 are in format of $mean \pm standard\ deviation$ obtained over various random splitting of cross-validation.

### Experiments on single omics data

**Table 1:** Mean square error (MSE) and coefficient of determination $R^2$ values for drug response prediction with single omics data

Dataset	Embedding	MSE	$\mathrm {R^2}$
mRNA expression	Dense	7.69 ± 4.05	−1.94 ± 1.76
	Graph	2.37 ± 0.16	0.24 ± 0.02
Mutations	Dense	11.41 ± 1.86	−3.42 ± 0.93
	Graph	3.25 ± 0.13	−0.02 ± 0.01
CNV	Dense	13.32 ± 2.23	−4.23 ± 0.93
	Graph	3.32 ± 0.14	−0.04 ± 0.03
RPPA expression	Dense	3.11 ± 0.19	−0.02 ± 0.05
metabolites	Dense	2.97 ± 0.13	0.07 ± 0.01

We first experimented with individual omics datasets and processed them in feedforward DNN. Table 1 shows results of drug response prediction with both mean squared error (MSE) and coefficient of determination $R^2$ [20]. MSE is used as our loss function for measuring the differences between predicted values and the true values. Let *i* and $n_c$ denote the index and the number of cell lines, and *j* and $n_d$ denote the index and number of drugs. MSE is calculated by

1 $$\begin{aligned} MSE(y,\hat{y}) = \frac{1}{n_c \times n_d} \sum _{j=1}^{n_d} \sum _{i=1}^{n_c} (y_{ij} - \hat{y}_{ij})^2 \end{aligned}$$

where $\hat{y}_{ij}$ refers to the predicted drug response value of cell line *i* and drug *j*, and $y_{ij}$ refers to the corresponding true value of their response. A model generally provides better predictive power when its MSE is smaller, and approaches perfect prediction when MSE is zero. However, MSE has no upper limits.

MSE is a straightforward metrics for regression model evaluation, whereas its assessment can be significantly influenced by the scale of the target value which is drug response level measured by $\log IC50$ in our experiments. In order to evaluate our models more fairly, we also used coefficient of determination $R^2$ which measures the proportion of output variables (i.e., drug responses) that is interpretable from the independent variables in the model [20]. $R^2$ is computed by

2 $$\begin{aligned} R^2(y,\hat{y}) = \frac{1}{n_d} \sum _{j=1}^{n_d} \left(1 - \frac{\sum _{i=1}^{n_c} (y_{ij} - \hat{y}_{ij})^2}{\sum _{i=1}^{n_c} (y_{ij} - \bar{y}_j)^2}\right) \end{aligned}$$

where $\bar{y}_j$ represents the average of response level of drug *j* across all cell lines. $R^2$ has a range of $[-\infty ,1]$ on the testing set. Unlike MSE, higher *R*^2^ indicates better model performance, and perfect prediction is achieved when *R*^2^ equals to one. When the model predicts all the outputs to be the average of true labels indiscriminately, *R*^2^ is zero. Moreover, it could also be negative occasionally, when the model performs even worse than producing averages all the time.

For mRNA expression, mutations, and CNV data, we compared the results with graph embeddings and dense embeddings. Graph embeddings allow the use of functional relationships of biomolecules from interactome data as prior information. As seen from the table, use of graph embedding clearly improved the MSE and $R^2$ values of drug response prediction from individual omics types. mRNA expressions gave the highest $R^2$ of 0.24 for drug response prediction.

### Experiments on multi-omics data

After measuring the individual models, we trained the models on whole dataset consisting of multi-omics types. During training the integrated model, the parameters in the embedding layer and dense layer were remained invariable, and only the attention layer’s parameters were learned. After removing the missing data points and keeping only the cell lines possessing all the omics features, our input dataset involved 522 cell lines in total. A three-fold cross-validation was implemented and the results of drug response prediction with fusion of all five-omics data are shown in Table 2. Individual omics types were processed by parallel DNN and combined using an attention layer. Attention layer improved both MSE and $R^2$ of prediction. The attention layer weighs omics layer embeddings learnt by DNN in a manner to improve prediction.

**Table 2:** Results of drug response prediction with multi-omics data

Model	MSE	$\mathrm {R^2}$
Multi-omics without attention layer	2.42 ± 0.22	0.65 ± 0.02
Multi-omics with attention layer	0.28 ± 0.01	0.90 ± 0.01

**Table 3:** Results of ablation experiments

Omics combination	MSE	$\mathrm {R^2}$
mut-rppa	0.26 ± 0.01	0.91 ± 0.01
exp-mut-rppa	0.27 ± 0.01	0.90 ± 0.01
exp-mut	0.27 ± 0.01	0.90 ± 0.01
mut-cnv-rppa	0.28 ± 0.01	0.90 ± 0.01
exp-mut-cnv-rppa	0.28 ± 0.01	0.90 ± 0.01
mut-rppa-metabolites	0.28 ± 0.01	0.90 ± 0.01
mut-cnv	0.28 ± 0.01	0.90 ± 0.01
exp-mut-rppa-metabolites	0.28 ± 0.01	0.90 ± 0.01
exp-mut-cnv	0.28 ± 0.01	0.90 ± 0.01
exp-mut-metabolites	0.28 ± 0.01	0.90 ± 0.01
exp-mut-cnv-rppa-metabolites	0.28 ± 0.01	0.90 ± 0.01
mut-metabolites	0.28 ± 0.01	0.90 ± 0.01
mut-cnv-rppa-metabolites	0.28 ± 0.01	0.90 ± 0.01
exp-mut-cnv-metabolites	0.28 ± 0.01	0.90 ± 0.01
mut-cnv-metabolites	0.28 ± 0.01	0.90 ± 0.01
exp-rppa	0.66 ± 0.04	0.78 ± 0.02
exp-cnv-rppa	0.67 ± 0.04	0.78 ± 0.02
cnv-rppa	0.67 ± 0.04	0.78 ± 0.02
exp-rppa-metabolites	0.67 ± 0.04	0.78 ± 0.02
exp-cnv-rppa-metabolites	0.67 ± 0.04	0.78 ± 0.02
rppa-metabolites	0.67 ± 0.04	0.78 ± 0.02
cnv-rppa-metabolites	0.67 ± 0.04	0.78 ± 0.02
exp-cnv	1.08 ± 0.05	0.65 ± 0.02
exp-cnv-metabolites	1.08 ± 0.05	0.65 ± 0.02
exp-metabolites	1.08 ± 0.05	0.65 ± 0.02
cnv-metabolites	1.84 ± 0.05	0.44 ± 0.03

To further explore the effectiveness of integrating different omics data, we performed ablation experiments by testing the performance of integrating all possible omics combinations. The results are displayed in Table 3, ranking in order of performance from better to worse. In the table, ’exp’, ’mut’, ’cnv’, ’rppa’ and ’metabolites’ refer to gene expression, gene mutations, CNV, RPPA and metabolites, respectively. It is shown that combinations of gene mutations and RPPA data achieved the highest performance. And apparently, when gene mutations are involved, the MSE is lower than 0.3 and $R^2$ is over 0.9 (as shown in the first 15 rows of Table 3). When Gene mutations are absent, but RPPA is still included, MSE rises to over 0.66 and $R^2$ decreases to around 0.78 (from row 16 to 22 of Table 3). If both gene mutations and RPPA are excluded, the performances become the worst as shown in the rest rows of Table 3.

**Fig. 1:**
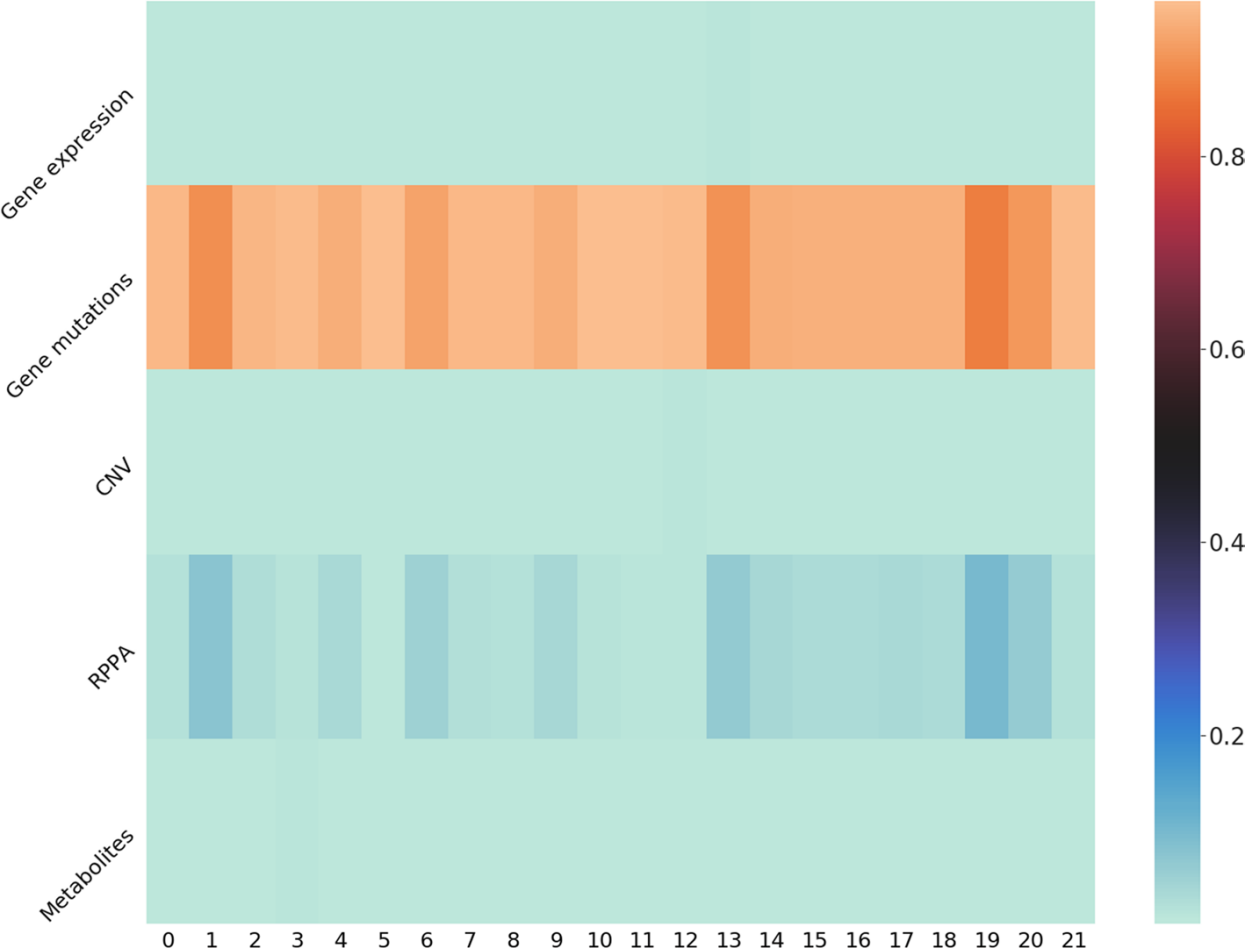
Attention scores on multi-omics of all the drugs. Gene mutation data obtain the highest attention score of around 0.9 over all drugs, and RPPA data obtain the second highest attention score of over 0.03. This observation conforms the findings of our ablation experiments, where Gene mutations and RPPA lead to better performances

Additionally, we preserved and analysed the attention weights of our models to demonstrate our findings above. Initially, the attention weights are in format of $(n_f \times m) \times n_{drug}$, where *m* denotes the number of omics and $n_f$ denotes the hidden size of output layer of sub-models regarding individual omics. Then the weights are summed across $n_f$ to be in format of $m \times n_{drug}$, representing the attention scores on each omics of all the drugs. We observed that when gene mutations data are involved, it always obtains a score around 0.9 over all the drugs, which means that it contributed about 90% of drug response prediction. And when gene mutation is excluded and RPPA is included, RPPA gains about 0.9 attention score of all drugs. An overall attention score heatmap is shown in Figure 1. Consequently, our analysis of attention weights is in accordance with the performances of ablation experiments, both of which illustrate that gene mutation and RPPA provide a greater predictive power towards drug responses than other omics.

### Prediction of top drugs

Table 4 shows the top 22 drugs ranked according to the MSE of prediction by the DNN, along with their targets and mechanism of action. As seen, the MSE differs largely between different drugs and can be used as network’s efficacy of response prediction. For some drugs like LGK974 and EPZ004777, our network was able to consistently predict relatively accurate drug responses whereas drugs like Trametinib and Camptothecin were predicted less accurately. Variable prediction accuracy may be attributed to different functional roles of drug targets and different biological mechanisms and efficacy through which drugs act.

**Table 4:** Drugs ordered according to the accuracy (MSE) of prediction

MSE	Drug name	Putative target	Pathway name
1.1	LGK974	PORCN	WNT signalling
1.2	EPZ004777	DOT1L	Chromatin histone methylation
1.4	EPZ5676	DOT1L	Chromatin histone methylation
1.4	GSK1904529A	IGF1R, IR	IGF1R signalling
1.5	MK-1775	WEE1, PLK1	Cell cycle
2.1	Palbociclib	CDK4, CDK6	Cell cycle
2.2	Afatinib	ERBB2, EGFR	EGFR signalling
2.3	PD0325901	MEK1, MEK2	ERK MAPK signalling
2.4	Linsitinib	IGF1R	IGF1R signalling
2.5	Oxaliplatin	DNA alkylating agent	DNA replication
2.6	Sapatinib	EGFR, ERBB2, ERBB3	EGFR signalling
2.6	PLX-4720	BRAF	ERK MAPK signalling
2.6	Alpelisib	PI3Kalpha	PI3K/MTOR signalling
2.7	SCH772984	ERK1, ERK2	ERK MAPK signalling
2.8	MK-2206	AKT1, AKT2	PI3K/MTOR signalling
2.8	Nutlin-3a (-)	MDM2	p53 pathway
3.2	5-Fluorouracil	Antimetabolite (DNA & RNA)	Other
3.7	Taselisib	PI3K (beta sparing)	PI3K/MTOR signalling
3.8	Irinotecan	TOP1	DNA replication
4.0	Luminespib	HSP90	Protein stability and degradation
4.1	Trametinib	MEK1, MEK2	ERK MAPK signalling
4.8	Camptothecin	TOP1	DNA replication

In order to evaluate the accuracy of our network further, we computed the rate at which the most effective drug predicted by our model in terms that the predicted drug response was in fact the recommended drug for that cancer cell type. To this end, we defined Top-1 and Top-3 candidates of drug predictions as the rates at which the true drug was predicted by our method as the topmost drug and within the top 3 drugs, respectively. Top-1 and Top-3 drug prediction accuracies of the network are given in Table 5. Since there are a total 22 drugs whose IC50 values were predicted, the chance of randomly choosing a drug that turns out to be the most effective drug is less than 5%. The best Top-1 accuracy of our single omics model is 50% achieved by gene expression and RPPA. And the best Top-3 accuracy is 91% achieved by RPPA. Compared to that, our multi-omics model performed quite well, increasing the chance of predicting the most effective drug up to 91%. The chance that the network predicted drug was among the actual top 3 most effective drug predicted drug improved close to 98%.

**Table 5:** Top-1 and Top-3 accuracies of prediction the desired drug

Omics type	Top-1 accuracy (%)	Top-3 accuracy (%)
Gene expression	50	89
Gene mutation	39	84
Gene CNV	39	84
Metabolomics	49	86
RPPA	50	91
Multi-omics (without attention)	51	87
Multi-omics (with attention)	91	98

### Comparison with existing methods

To demonstrate the effectiveness of our approach, we compared it with Bayesian multitask multi-kernel learning (BEMKL), proposed by Ali et al. [21]. BEMKL was the winning method on DREAM 7 challenge in 2014 [22], and in 2018 the authors further extended this method to adapt multi-omics data input. The major idea of this approach is to estimate kernels for each omics and integrate the kernels via multi-view learning. This method originally takes Gene expression, Gene mutations, CNV, Mass Spectrometry, and miRNA expression as inputs. To compare with our approach on the same dataset, Mass Spectrometry and miRNA expression were replaced by RPPA and Metabolomics data. As shown in Table 6, although MSE and $R^2$ are approximate on single omics’ condition, our method significantly outperforms BEMKL in the case of multi-omics.

We compared our approach with DeepDR [8], a deep learning approach proposed for drug response prediction. DeepDR utilizes an auto-encoder to learn latent representations from gene expressions and gene mutation data and then merges the learnt features together for prediction. Our work is similar to DeepDR from the perspective of two-stage training and latent features learning whereas our work is capable of integrating multiple types of omics data and leveraging an attention mechanism to weigh their influences efficiently. The hyper-parameters of DeepDR model followed the same way as in [8] since they were also tuned on the same CCLE dataset. As seen in Table 7, our method achieves a lower MSE and a higher $R^2$. These comparisons illustrate that, with the attention layer, our model is capable of integrating multi-omics data effectively considering their distinct interactions in various drugs.

**Table 6:** Performance comparison with Bayesian multi-task multi-kernel learning (BEMKL) method

Dataset	Our method		BEKML	
	MSE	$\mathrm {R^2}$	MSE	$\mathrm {R^2}$
Gene expression	2.37	0.24	2.37	0.31
Gene mutations	3.25	−0.02	3.31	0.02
CNV	3.32	−0.04	4.51	−0.22
RPPA	3.11	−0.02	2.52	0.33
Metabolites	2.97	0.07	3.25	0.10
Multi-omics	0.46	0.84	2.32	0.34

**Table 7:** Performance comparison with DeepDR

	MSE	$\mathrm {R^2}$
Our work	0.28 ± 0.01	0.90 ± 0.01
DeepDR	3.02 ± 0.17	0.10 ± 0.02

## Discussion

In this work, we investigated prediction of cancer cell lines’ responses to already available anti-cancer drugs by integrating deep learning and multi-omics approaches. The ability to predict drug response of a drug from a patient’s omics data enable drug repurposing and personalized treatment against cancer. We developed a DNN model capable of handling large and complex multi-omics data and integrate heterogeneous multi-omics information for drug response prediction. Our model was able to integrate heterogeneous multiple omics data effectively and to predict the most effective drugs and their activity against the patient’s specific cancer. Using our DNN model, we were able to achieve an $R^2$ value of drug response prediction and 98% accuracy of Top-3 drug prediction accuracy.

There are numerous challenges in the implementation of DNN for drug response prediction. These include huge dimensionality of inputs, heterogeneity of inputs as data come from different omics platform, limited number of samples, and a requirement for large number of network parameters. This culminates in a need for a large number of samples for adequate training of deep neural networks. Previous attempts with DNN were limited to only two types of omics data and our model offer a general strategy to integrate any number of omics data types. In order to handle high dimensionality of omics data types, we proposed two embedding strategies - graph embedding and dense embedding. Furthermore, we demonstrated how graph embeddings enabled incorporation of interactome data. The attention layer offered an efficient mechanism for combining information from different omics data type. We employed attention mechanism only at the final layer. One can explore how attention mechanism can be employed at hidden layers as well. It is also noteworthy to explore how the number of trainable parameters can be further reduced. With decreasing cost of collecting omics data, there is need for novel computational techniques that can effectively integrate multi-omics data for downstream tasks such as personalized diagnosis and treatment.

In a recent survey done to compare methods for drug prediction in cancer line few deep neural networks were featured in the comparative study [23]. In this review the CDRscan a deep neural network method that was used did not perform as well as Bayesian methods or Matrix factorization methods, therefore it was concluded that the implementation of deep learning neural networks was non-trivial and requires extensive optimisation. Therefore, in the above investigation we seek to optimize the implementation of deep learning neural networks so that their performance in drug prediction improves and surpass other machine learning methodology.

## Conclusion

There were two novelties of our DNN model: (i) incorporation of functional interactions for processing mRNA expressions, gene mutations and CNV data, and (ii) use of an attention layer for combining embedding learned by networks of individual omics data. Our experiments show that the interactome data in the form of protein-protein interactions (PPI) improves drug response predictions. PPI data represents prior information among genes/proteins and explicit embedding of PPI significantly improves drug response prediction. The attention mechanism learns the weights for different omics data and improves the prediction accuracy. Our network outperforms feedforward neural networks without using graph embeddings or the attention layer.

In addition, the observations from the ablation experiments and the attention score distribution reveal that, among the multi-omics data, Gene mutation data distinctly contributes more to the prediction of drug responses than other omics data. When Gene mutation is excluded, RPPA dominates the weight of prediction. Therefore, our proposed approach provides a great predictive power of anti-cancer drug responses, together with an insight of the potential reactions between cell lines and drugs.

## Methods

### Datasets

We downloaded mRNA expressions, genomic mutations, reverse phase protein array (RPPA) expressions, and metabolomics data from CCLE, and copy number variations (CNV) and half maximal inhibitory concentration (IC50) of drug responses from GDSC, and interactome data from HINT^1^ (High-quality INTeractomes) database. The details of the datasets used in the experiments are summarized in Table 8.

**Table 8:** Details of omics datasets used

Source	Dataset	Size
CCLE	mRNA expressions	1210 cell lines, 19,145 genes
	gene mutations	1656 cell lines, 18,789 genes,1,239,235 mutations
	RPPA expressions	899 cell lines, 215 proteins
	metabolomics data	928 cell lines, 227 metabolites
GDSC	CNV data	987 cell lines, 25,638 genes
	IC50 responses	810 cell lines, 175 drugs
HINT	Interactome	62,345 binary protein pair interaction

- *mRNA expressions*: mRNA expression profiles in cells are measured by RNA sequencing technique in transcripts per million (TPM), which indicate how many genes actively transcribed in the cell. Earlier, analysis of RNA-seq data has revealed a comprehensive portrait of gene expressions in these cell lines [24] and contributions of deviations of expression level from the norm for certain genes to cancer, for example, see [25] for neuroblastoma. In this research, RNA-seq data of 1,210 cell lines and 19,145 genes were downloaded from CCLE.
- *Gene mutations*: Gene mutations involve numerous variations, such as silent, missense, nonsense, deletion, insertion, splice site, nonstop, etc. Cancer development usually involves the accumulation of multiple gene mutations [26]. For example, somatic mutations which fare relatively stable lead to initiation and progression of breast cancer [27]. We downloaded Gene mutation data including mutation position and variation for 1,656 cell lines and for 18,789 genes from CCLE.
- *Copy number variations (CNV)*: Copy Number Variation (CNV) records the number of copies of a gene in a cell and are structurally variant regions in which copy number differences have been observed between two or more genes, which are highly characteristics of cancer [28]. CNV have been believed to be highly correlated to differential gene expressions [29]. CNV data for 25,638 genes in 987 cell lines were downloaded from GDSC.
- *RPPA data*: Reverse Phase Protein Array (RPPA) is a high-throughput proteomics method that, comparing to mass spectrometry proteomics, has higher sensitivity for low-abundance proteins [30] and provides the expression data for a prespecified set of proteins. RPPA captures the state of key signal transduction pathways in cells and provides insights to the cancer mechanisms [31]. RPPA data that consists of protein expression level of 215 proteins in 899 cell lines were downloaded from CCLE.
- *Metabolomics data*: Metabolomics involves study of small-molecule biochemicals (metabolites) within a biologic system [32]. Certain metabolites have been validated as cancer biomarkers in various patient samples including blood, urine, and prostatic secretions [32]. Metabolomics directly reflect the underlying biochemical activities of the cells and are therefore very useful in cancer research. We downloaded the expression level of 227 metabolites in 928 cell lines from CCLE.
- *Interactome data*: Interactome data such as protein-protein interaction (PPI) lend us an overview of interactions among biological processes. HINT database [33] consists of a curated compilation of high-quality protein interactions from 8 interactome resources: BioGRID, MINT, iRefWeb, DIP, IntAct, HPRD, MIPS and the PDB. We downloaded interactome data with 62,345 binary protein pair interactions from HINT. The values in the PPI matrix were binary: 1 for an interaction, or 0 otherwise.
- *Drug response data*: In the experiments of this research, IC50 values (the half maximal inhibitory concentration) were adopted to evaluate drug response levels. IC50 values were measured in uM and represented in log scale where lower the IC50 value, more effective the drug [34]. We downloaded IC50 values measuring drug response from 810 cell lines against 175 anticancer drugs, and top 22 drugs were chosen because they were the most commonly used drugs and tested on more than 95% of the cell lines.

### Neural network architecture

**Fig. 2:**
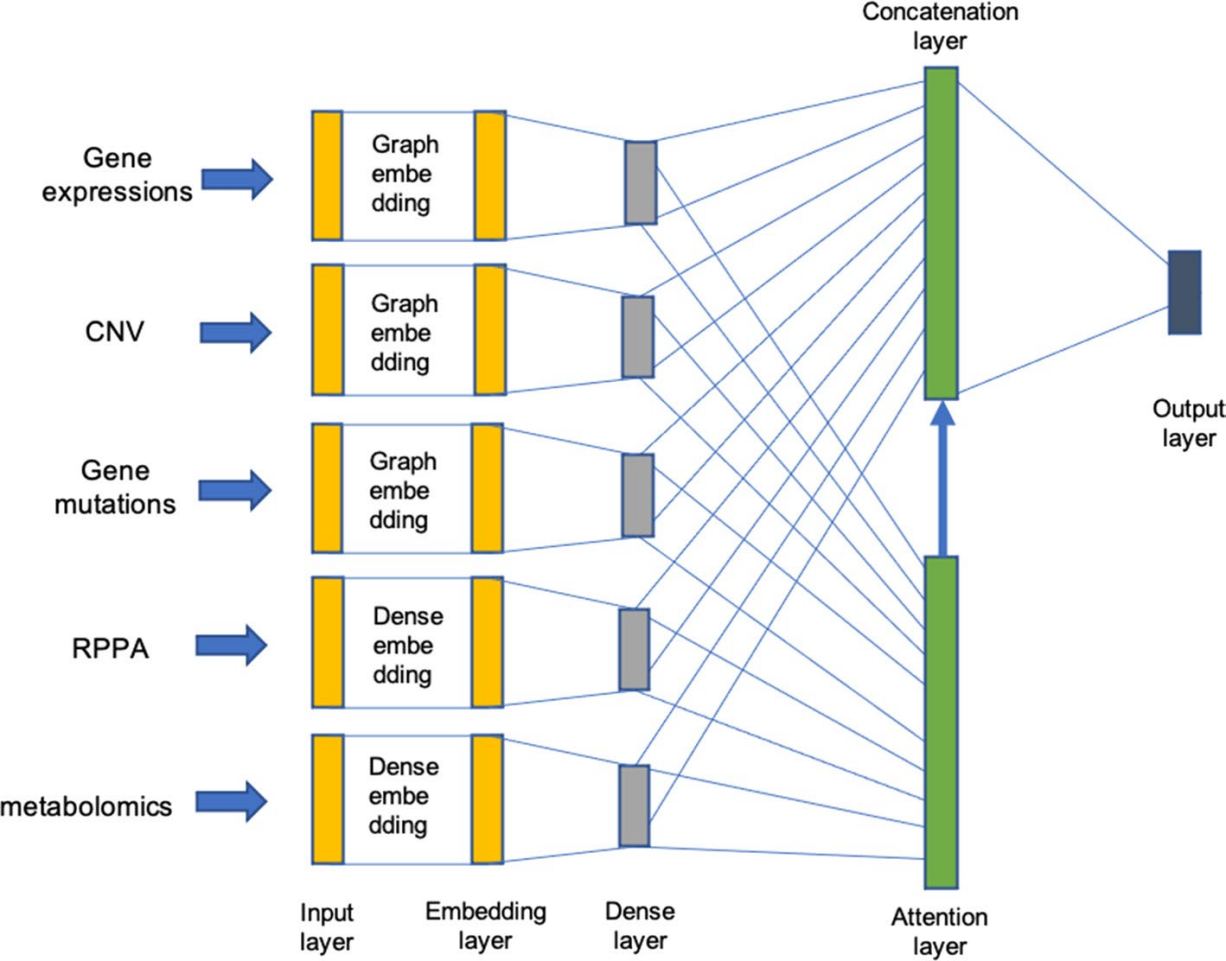
Deep neural network architecture combining different omics data. Gene expressions, CNV, and Gene mutations data are fed into a graph embedding layer, respectively, whereas RPPA and metabolomics data are fed into a dense embedding layer, respectively. In a graph embedding layer, information about interactions among genes obtained from HINT database is incorporated, and only genes with mutual interactions are distilled. Then a dense layer is applied for further learning the latent features of each omics dataset. Eventually, an attention layer is adapted to predict the final drug responses with distinct attention to different omics features

Figure 2 illustrates the deep neural network (DNN) architecture combining different omics data. The input to the network consists of 5 omics data types: mRNA expression, mutations, CNV, RPPA expressions, and metabolomics. Let the network input $x=\{x_m\}_{m=1}^M$ be a set of M omics datasets and $x_m=(x_{mi})_i^{n_m}$ denote the $m^{th}$ omics data consisting of $n_m$ features. The network consists of 3 hidden layers: an embedding layer ($l=1$), a dense layer 2 ($l=2$), and an attention layer ($l=3$). Let $y^l=\{y_m^l\}$ denote the $l^{th}$ hidden layer output and for $y_m^l$ denote the output due to $m^{th}$ omics data.

In order to incorporate the prior information from the interactome, embedding layer for mRNA expressions, mutations, CNV, and RPPA expressions used graph embedding while the metabolomics data goes through a dense embedding. The output of dense embedding layer is given by

3 $$\begin{aligned} y_m^1=f({(W_m^1)}^T x_m+b_m^1) \end{aligned}$$

and the output of graph embedding layer [18] is given by

4 $$\begin{aligned} y_m^1=f({(W_m^1 \cdot A)}^T x_m+b_m^1) \end{aligned}$$

where A denotes the affinity matrix of the protein-protein interaction network and $\cdot$ denotes element-wise product. $W_m^1$ and $b_m^1$ denote the weight matrix and bias vector of the embedding layer for $m^{th}$ omics data. The bias vector of this layer is initialized as zeros and learned during the training process.

The graph embedding layer allows incorporating prior information about interactions among genes/proteins, underlying fundamental biological mechanisms of cancer [18]. From the HINT database, we obtained the binary interactome dataset which records pairwise protein-protein interactions. The graph embedding layer acts as a feature filter that prevents information from proteins/genes that do not have biological interactions with others from being fed into its corresponding hidden neurons, therefore achieving our goal of a sparse connection. Since the interactions between metabolites are unknown, we used dense embedding for metabolomics data.

The embedding layer is followed by one dense layer. The output of the dense layer for m omics data is given by

5 $$\begin{aligned} y_m^2=f({(W_m^2)}^T y_m^1+b_m^2) \end{aligned}$$

where $W_m^2$ and $b_m^2$ denote the weight matrix and bias vector for $m^{th}$ omics data for dense layer. The bias vector of this layer is initialized as zeros and learned during the training process.

The dense layer outputs of different omics data are concatenated as z:

6 $$\begin{aligned} z=[y_m^2]_{m=1}^5 \end{aligned}$$

Assume that the attention layer weight $W^3$ is composed of ${W^3 (d,m,i)}$, where $W^3(d,m,i)$ represents the weight corresponding to drug *d*, dataset *m*, and feature *i*. Output layer weights *W*(*d*, *m*, *i*) are defined by

7 $$\begin{aligned} W(d,m,i)=\frac{e^{W^3(d,m,i)} \cdot k(d,i)}{\sum _m e^{W^3(d,m,i)}} \end{aligned}$$

where $\{ k(d,i) \}$ denotes the kill matrix used for enforcing output neurons in the final layer to focus on its corresponding neurons from each individual omics sub-network. Specifically, we maintain the hidden sizes of the dense layers of all omics in Figure 2 to be the same with final output size, which means the range of *d* and *i* will be the same. Then the *k*(*d*, *i*) can be designed to be 1 when $d=i$, or 0 when $d\ne i$. Element-wise multiplication of natural exponential of weights and the kill matrix ensures that the output neuron representing a drug solely focuses on the corresponding neurons in preceding sub-networks that represents the same drug, and influences coming from other neurons are set to zero. This design prevents prediction of one drug being affected by other drugs.

The output *y* is given by

8 $$\begin{aligned} y=g(W^Tz+b) \end{aligned}$$

## Data Availability

Data used in this study can be downloaded from CCLE and GDSC database.

## Footnotes

1 http://hint.yulab.org/http://hint.yulab.org/
